# Supplementary material for: Structural and functional evidence of bacterial antiphage protection by Thoeris defense system via NAD+ degradation
Source: Nat Commun. 2020 Jun 4;11:2816. doi: 10.1038/s41467-020-16703-w (PMC7272460; doi:10.1038/s41467-020-16703-w)
Supplement: Supplementary file 1 — Supplementary Information [file 41467_2020_16703_MOESM1_ESM.pdf]

Supplementary Information

Structural and functional evidence of bacterial antiphage protection by Thoeris defense system via NAD^+ degradation

Donghyun Ka, Hyejin Oh et al.

Supplementary Table 1. Data collection, phasing and refinement statistics[a].

	ThsA SeMet	ThsB SeMet
Space group	C2	$P2_1$
Unit cell parameters (Å)	a=180.4, b=168.6, c=93.6 β=106.71°	a=53.6, b=67.7, c=59.2 β=92.4°
Solvent content (%)	61.4	47.9
Wavelength (Å)	0.9792	0.9793
**Data collection statistics**		
Resolution range (Å)	50.00–2.50 (2.59–2.50)	50.00–1.80 (1.86–1.80)
Number of reflections	91771 (9063)	77256 (7689)
Completeness (%)	99.6 (99.1)	99.9 (99.9)
R_{merge}[b]	0.140 (1.666)	0.092 (0.605)
CC1/2	0.990 (0.543)	0.941 (0.761)
CC*	0.997 (0.839)	0.985 (0.930)
Redundancy	7.3 (7.2)	3.8 (3.8)
Mean I/σ	10.5 (1.1)	12.3 (2.3)
**Phasing statistics**		
Number of Se sites (found/possible)	27/32	2/4
Figure of merit	0.27	0.36
**Refinement statistics**		
Resolution range (Å)	47.81-2.50	29.56-1.80
R_{cryst}[c]/R_{free}[d] (%)	24.4/27.2	19.3/22.6
RMSD bonds (Å)	0.003	0.007
RMSD angles (deg)	0.81	0.95
Average B-factor, Protein/Water ($Å^2$)	82.2/54.6	28.1/33.1
Number of water molecules	36	332
Ramachandran favored (%)	96.6	98.5
Ramachandran allowed (%)	3.4	1.5

[a]Values in parentheses are for the highest-resolution shell.
[b]$R_{merge} = \Sigma_h \Sigma | I_i(h) - \langle I(h) \rangle | / \Sigma_h \Sigma_i I_i(h)$, where $I_i(h)$ is the intensity of an individual measurement of the reflection and $\langle I(h) \rangle$ is the mean intensity of the reflection.
[c]$R_{cryst} = \Sigma_h || F_{obs} | - | F_{calc} || / \Sigma_h | F_{obs} |$, where F_{obs} and F_{calc} are the observed and calculated structure factor amplitudes, respectively.
[d]R_{free} was calculated as R_{cryst} using ~5% of the randomly selected unique reflections that were omitted from structure refinement.

Supplementary Table 2. RMSD values of the Cα atomic positions between the N-terminal domain of ThsA and sirtuin structures.

Sirtuin protein	Organism	PDB ID	RMSD (Å)	No. of aligned residues
CobB	*Thermotoga maritima*	2H2G	3.3	159
CobB1	*Archaeoglobus fulgidus*	4TWI	2.9	177
Hst2	*Saccharomyces cerevisiae*	1Q14	3.6	171
Sirt2	*Homo sapiens*	1J8F	3.6	184
Sir2A	*Plasmodium falciparum*	3U3D	2.7	172
Sirt3	*Homo sapiens*	3GLS	3.4	171
Sirt4	*Xenopus tropicalis*	5OJ7	3.6	172
Sirt5	*Danio rerio*	4UTN	3.0	163
Sirt5	*Homo sapiens*	4F4U	3.3	174
Sirt6	*Homo sapiens*	3K35	3.6	147

Supplementary Table 3. Top ten structural neighbors of the TIR domain (the Rossmann-like fold portion) of ThsB identified by the Dali server[1].

Protein	Organism	PDB ID	Z score	RMSD (Å)	No. of aligned residues
TIR domain of the Toll-receptor-related 2 (TRR-2)	*Hydra vulgaris*	4W8G	13.3	2.2	104
TIR domain of the Sterile alpha and TIR motif-containing protein 1 (SARM1)	*Homo sapiens*	6O0U	12.8	2.5	105
2'-deoxynucleoside 5'-phosphate N-hydrolase 1 (Dnph1)	*Rattus norvegicus*	4FYI	12.1	3.1	106
TIR domain of the disease resistance protein, Recognition of Peronospora parasitica 1 (RPP1)	*Arabidopsis thaliana*	5TEB	10.8	3.1	100
Toll/interleukin-1 receptor domain-containing adapter protein (TIRAP)	*Homo sapiens*	5UZB	11.4	2.7	107
TIR-NB-LRR type resistance protein, RPV1	*Vitis rotundifolia*	5KU7	10.8	3.3	104
TIR domain of the SUPPRESSOR OF npr1-1, CONSTITUTIVE 1 (SNC1)	*Arabidopsis thaliana*	5H3C	10.5	3.4	99
Nucleoside 2'-deoxyribosyltransferase*	*Bacillus psychrosaccharolyticus*	6EVS	11.1	2.6	101
2'-deoxynucleoside 5'-phosphate N-hydrolase 1 (Dpph1)	*Homo sapiens*	4P5D	11.1	3.1	103
Small GTP-binding protein, HydF*	*Thermosipho melanesiensis*	5KH0	10.9	2.6	99

*Bacterial proteins.

Supplementary Table 4. Primers used in this study.

Primer		5′-3′ sequence	Description
$(His)_6$-MBP-ThsA	Forward	AGGGCCATATGAATCCTATCGTGGAGCTGTTTATTAAGG	Cloning into pET28a vector
	Reverse	TGGTGCTCGAGTTAGTTGGAAAGAATTTCCACAAACTC	
$(His)_6$-MBP-ThsB	Forward	ATTTCCAGGGCCATATGGCGAAACGGGTCTTTTTC	Cloning into pET28a vector
	Reverse	GGTGGTGGTGCTCGAGTTAGTTTACCCAGGAC	
ThsA N112A	Forward	CACACATACTGGACAACCGCCTATGATAGATTAATAG	Site-directed mutagenesis
	Reverse	CTATTAATCTATCATAGGCGGTTGTCCAGTATGTGTG	
ThsA H152A	Forward	GCTGTTGTGTATAAGATGGCCGGTGATGTTGAGCATC	Site-directed mutagenesis
	Reverse	GATGCTCAACATCACCGGCCATCTTATACACAACAGC	
Untagged ThsB	Forward	ATTTCCAGGGCCATATGGCGAAACGGGTCTTTTTC	Cloning into pET21a vector
	Reverse	GGTGGTGGTGCTCGAGTTGGCACTAGTTTACCCAGGAC	

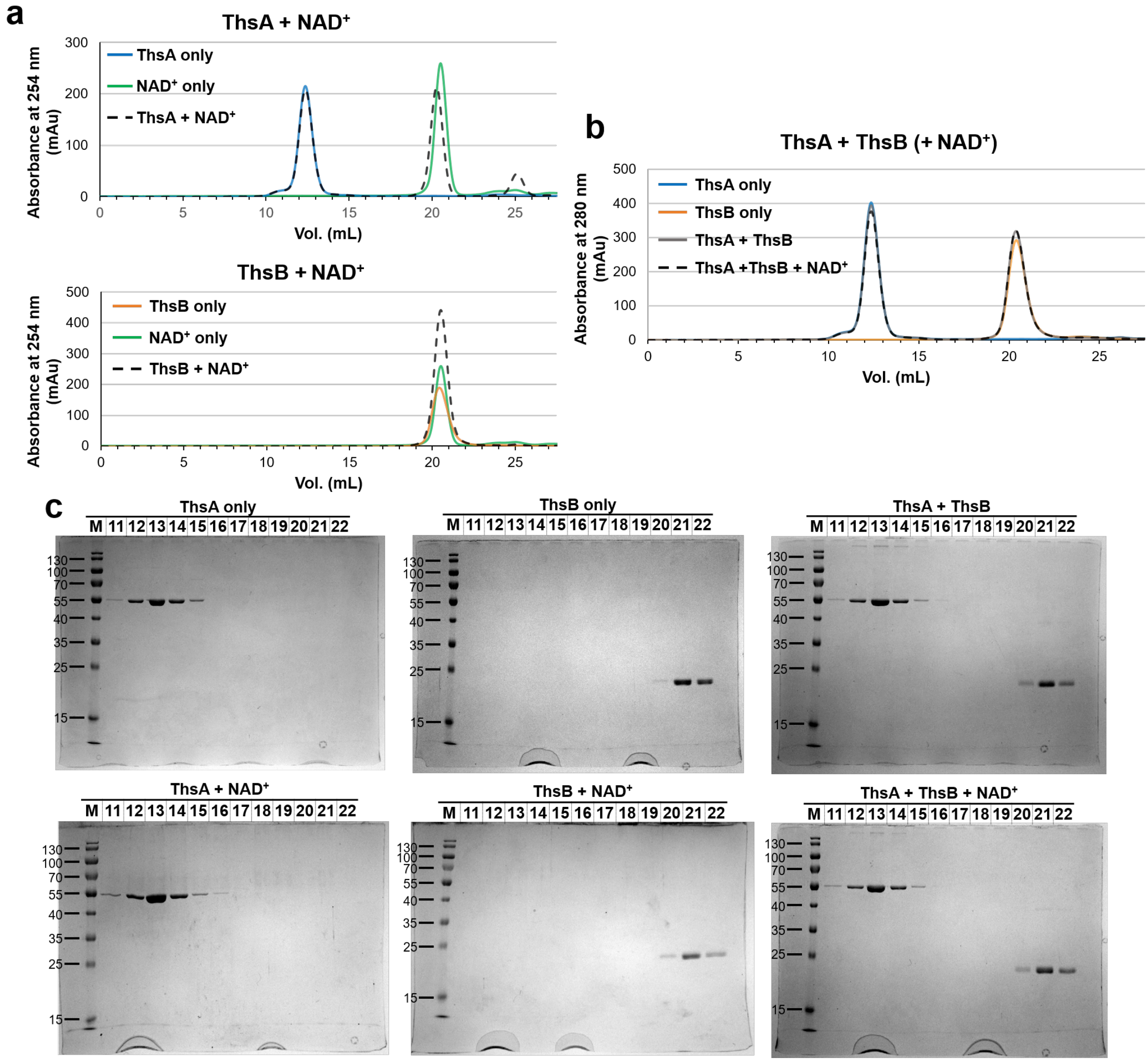

Supplementary Fig. 1. Analytical SEC for testing the interactions between Thoeris proteins and NAD$^+$. a, SEC analysis of individual Thoeris proteins with NAD$^+$. The chromatographic peak of NAD$^+$ was divided into two parts in the presence of ThsA. Absorbance was measured at 254 nm to detect both NAD$^+$ and Thoeris proteins. An identical 'NAD$^+$ only' chromatogram (green line) is shown twice as a control for comparison. **b**, SEC analysis for testing the complex formation of ThsA and ThsB with and without NAD$^+$. ThsB does not form a complex with ThsA. Absorbance was measured at 280 nm. **c**, Gel images from sodium dodecyl sulfate–polyacrylamide gel electrophoresis for the SEC elution fractions. Source data are provided as a Source Data file.

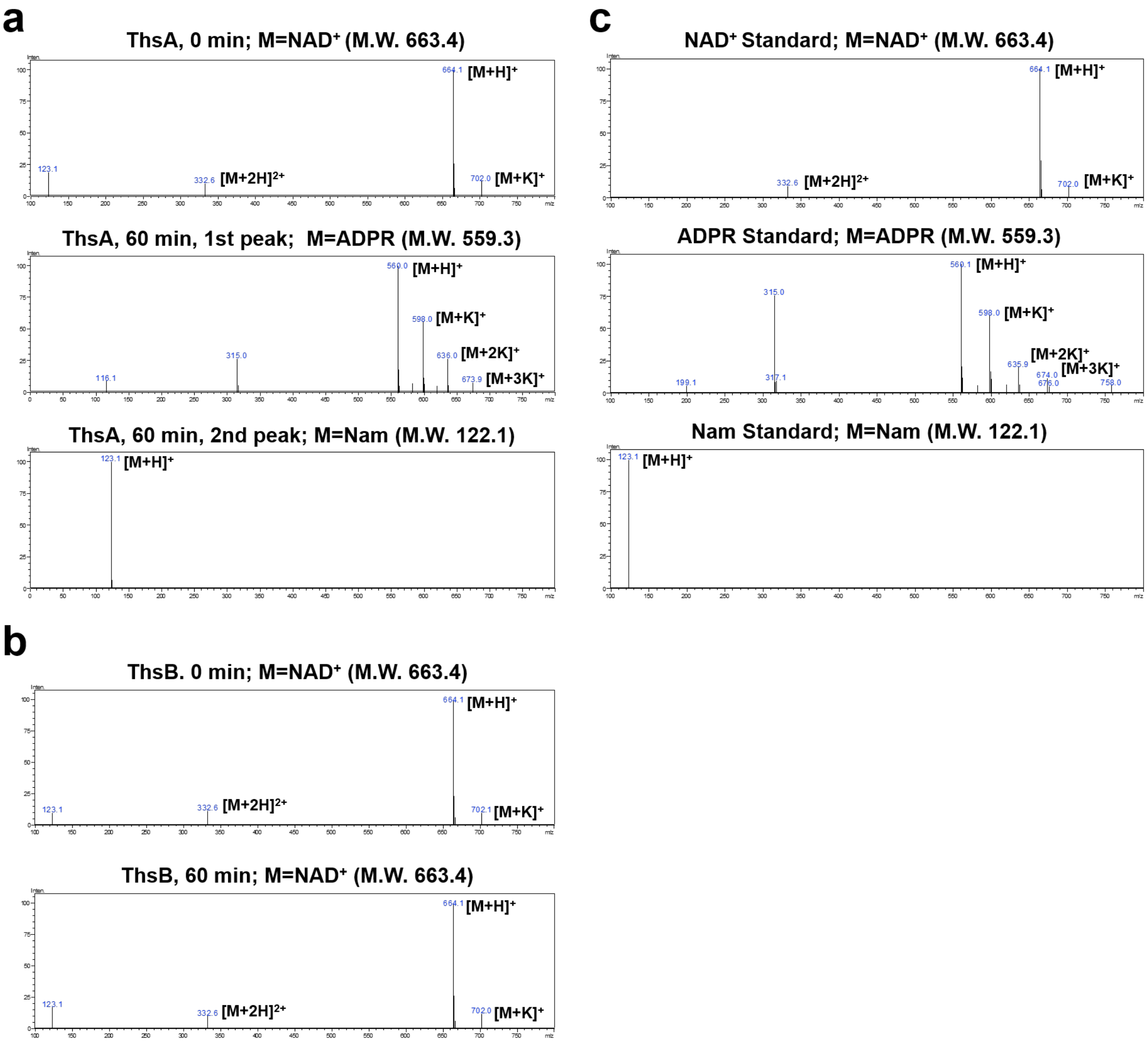

Supplementary Fig. 2. MS analyses of NAD$^+$ cleavage products. a-c, MS spectra of the LC chromatographic peaks from the NAD$^+$ cleavage assay of ThsA (**a**), ThsB (**b**), and controls with standard compounds (NAD$^+$, Nam, and ADPR; **c**) in Fig. 1b. The peaks for protonated molecules ([M+H]$^+$) and adduct ions ([M+nK]$^+$ or [M+2H]$^+$) are annotated. All peaks in the MS spectra are shown relative to the value of the most abundant ion (base peak) set to 100%.

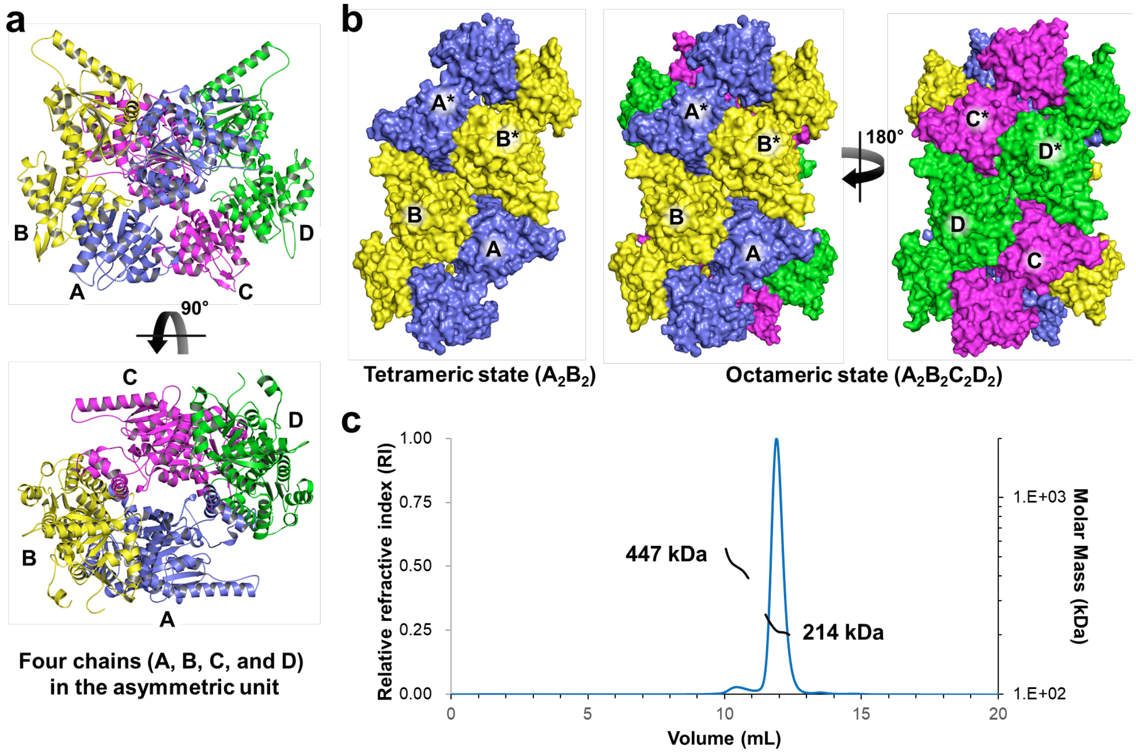

Supplementary Fig. 3. Oligomeric state of ThsA. a, Four ThsA protomers in the asymmetric unit of the crystal structure. **b**, Putative quaternary structures of ThsA. Tetrameric and octameric states were predicted by PISA[2]. The asterisks indicate symmetry-related (-x, y, -z) molecules. **c**, SEC-MALS analysis of ThsA. The average molecular masses corresponding to the two peaks are consistent with an octamer and a tetramer, whose theoretical molecular weights are 441.0 kDa and 220.5 kDa, respectively. Source data are provided as a Source Data file.

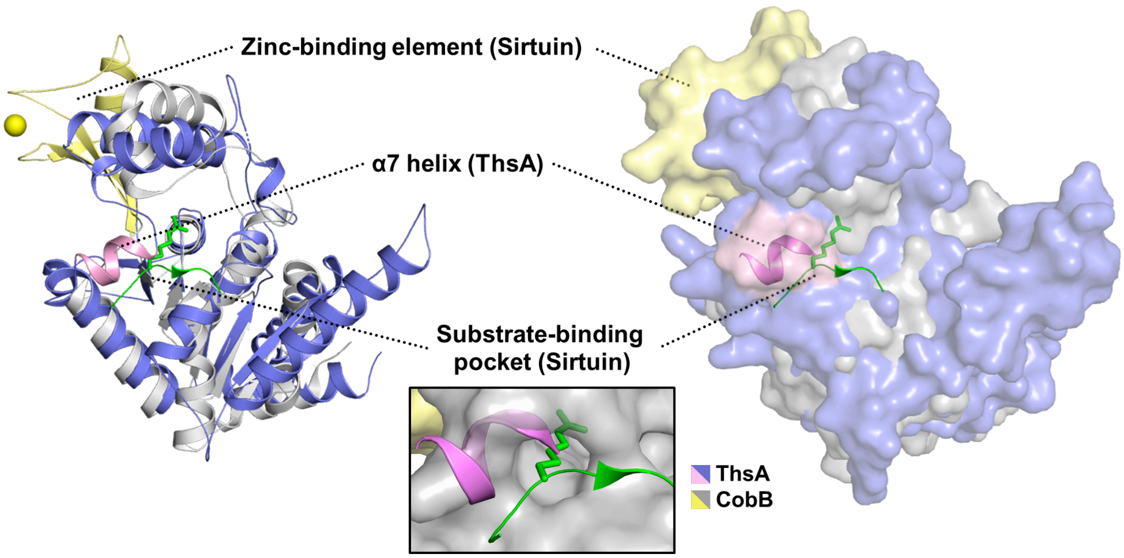

Supplementary Fig. 4. Structural differences between the N-terminal domain of ThsA and sirtuins. The N-terminal domain of ThsA (blue) is structurally aligned with a sirtuin protein, *T. maritima* CobB (PDB ID: 2H2F; grey). The acetyl substrate-binding pocket of sirtuins is not available in the N-terminal domain of ThsA due to blocking by its α7 helix (pink). The zinc-binding element (yellow) in the *T. maritima* CobB structure is highlighted with the essential zinc ion (a yellow sphere). The bound substrate, a histone H3 peptide with N6-acetyllysine, is also shown in green.

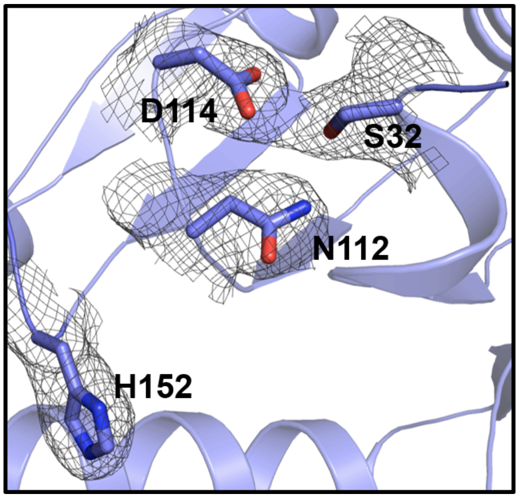

Supplementary Fig. 5. Electron density map of key residues in the putative NAD^+ binding site of ThsA. The $2mF_{obs} - DF_{calc}$ map is contoured at 1.0 σ.

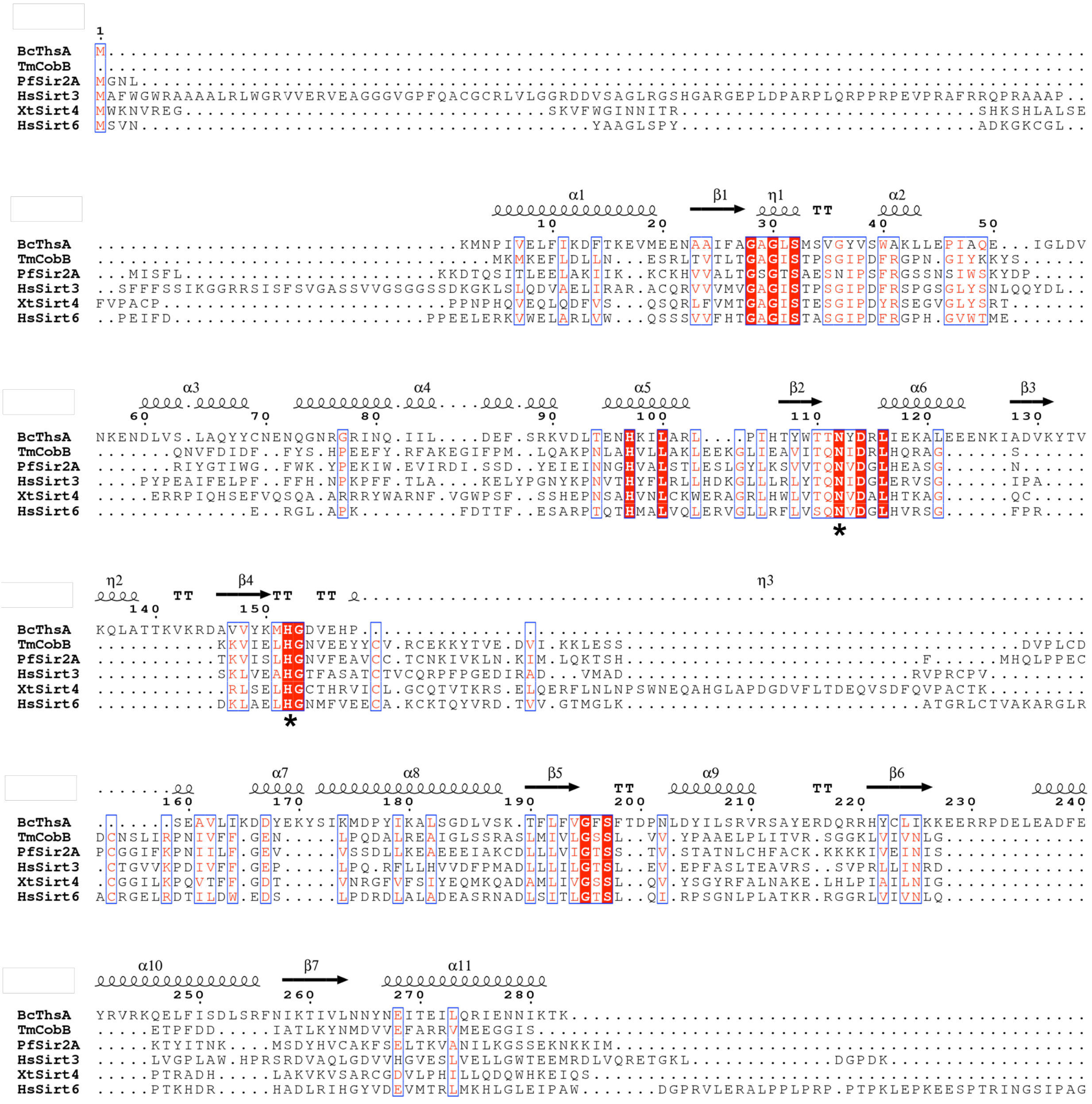

Supplementary Fig. 6. Sequence alignment of the N-terminal domain of ThsA with sirtuin proteins. Asn112 and His152 of ThsA, whose roles were examined by mutation analysis, are marked with asterisks. White character in red box indicates strict identity. Red character and blue frame represent similarity in a group and across groups, respectively. Bc, *B. cereus*; Tm, *T. maritima*; Pf, *Plasmodium falciparum*; Hs, *Homo sapiens*; Xt, *Xenopus tropicalis*.

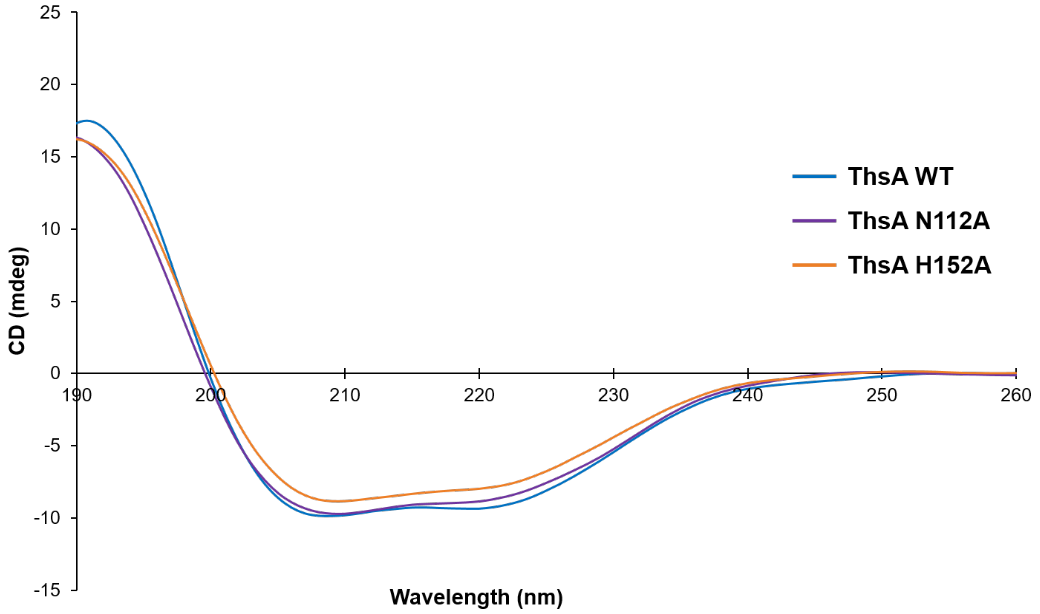

Supplementary Fig. 7. CD spectroscopy for wild type and mutant ThsA proteins. All the proteins exhibited CD spectra that are characteristic of folded proteins. Source data are provided as a Source Data file.

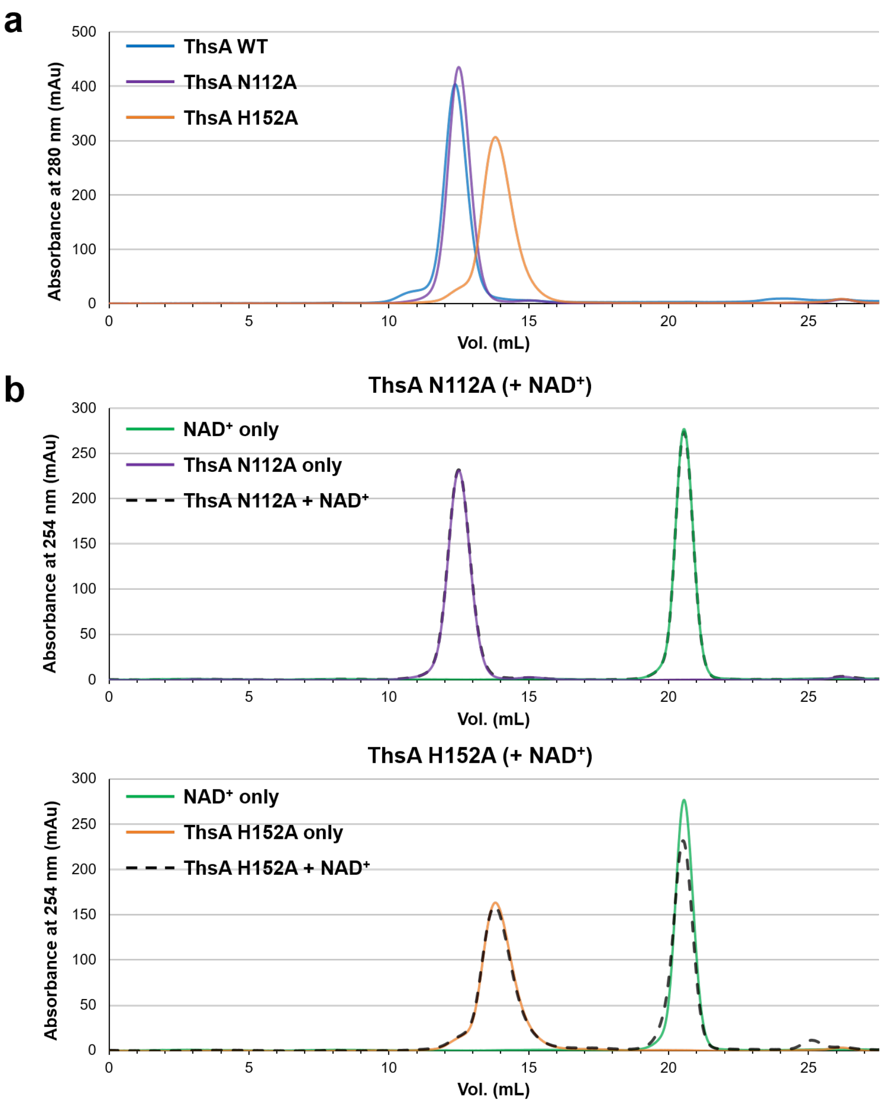

Supplementary Fig. 8. Analytical SEC for mutant ThsA proteins. a, SEC analysis of individual ThsA mutants. Absorbance was measured at 280 nm. The chromatogram for the wild type ThsA (Supplementary Fig. 1b) is shown again as a control for comparison. The retention volume of the N112A mutant was essentially identical to that of the wild type ThsA, whereas the H152A mutant was eluted significantly later, indicating difference in the oligomeric state. **b**, SEC analysis for testing NAD^+ binding of ThsA mutants. Absorbance was measured at 254 nm to detect both NAD^+ and proteins. The 'NAD^+ only' chromatogram (Supplementary Fig. 1a) is shown again as a control for comparison. Neither of the two ThsA mutants bound to NAD^+. Source data are provided as a Source Data file.

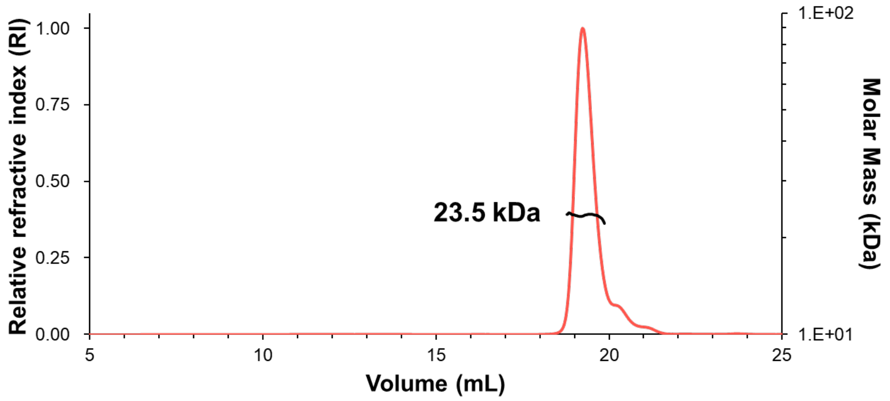

Supplementary Fig. 9. ThsB exists as a monomer in a solution. The oligomeric state of ThsB was investigated using SEC-MALS. The average molecular mass corresponding to the peak is consistent with a monomer, whose theoretical molecular weight is 22.5 kDa. Source data are provided as a Source Data file.

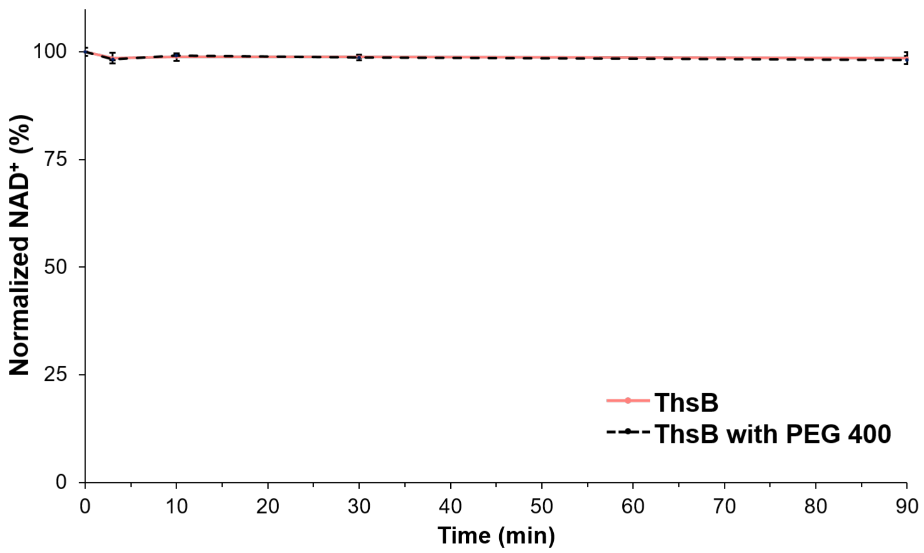

Supplementary Fig. 10. Effect of a macromolecular crowding agent on NAD^+ cleavage by ThsB. Addition of 15% (w/v) PEG 400 did not stimulate NAD^+ degradation by ThsB. For comparison, the result of ThsB without the macromolecular crowding agent in Fig. 1a is shown again. Data are presented as mean ± s.e.m. for three independent experiments. Source data are provided as a Source Data file.

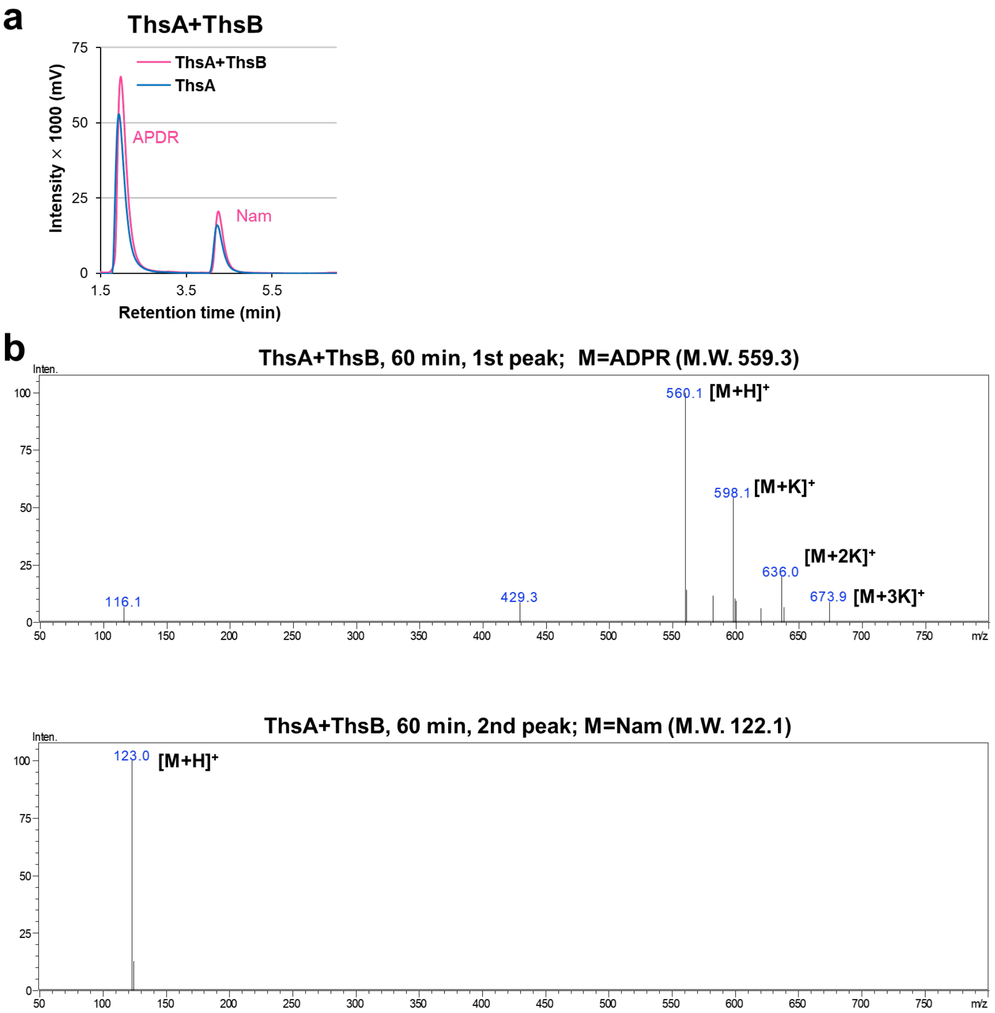

Supplementary Fig. 11. ThsB does not alter NAD$^+$ cleavage function of ThsA or further degrade NAD$^+$ cleavage products. a, LC analysis of the NAD$^+$ cleavage reaction in the presence of both ThsA and ThsB. Products of this reaction generated a chromatogram (magenta line) essentially identical to that of the only ThsA-added reaction (blue line). This indicates that, in the presence of ThsB, ThsA is still functional, and the cleavage products (Nam and ADPR) remain intact. For comparison, the LC result of ThsA in Fig. 1b is shown again. Source data are provided as a Source Data file. **b**, MS spectra of the NAD$^+$ cleavage products generated in the presence of both ThsA and ThsB. The first and second chromatographic peaks in **a** were identified as ADPR and Nam, respectively.

a

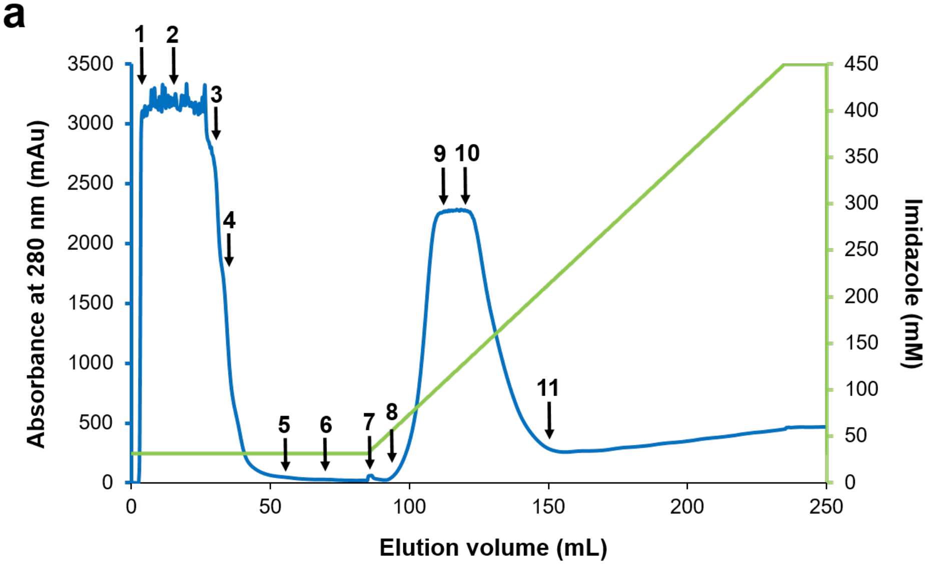

b

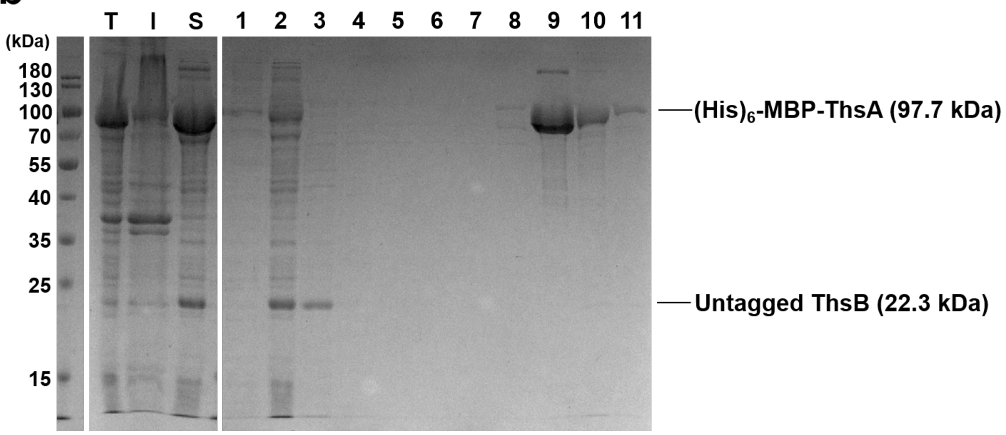

Supplementary Fig. 12. Analysis of co-expressed Thoeris proteins. a, Purification of co-expressed Thoeris proteins. (His)$_6$-MBP-tagged ThsA was expressed together with untagged ThsB in *E. coli* cells, and purified using nickel-affinity chromatography. **b,** Gel electrophoretic analysis of co-expressed Thoeris proteins. Total (T), insoluble (I) and soluble (S) expressed proteins and fractions from the nickel-affinity chromatography were analyzed by SDS-PAGE. Source data are provided as a Source Data file.

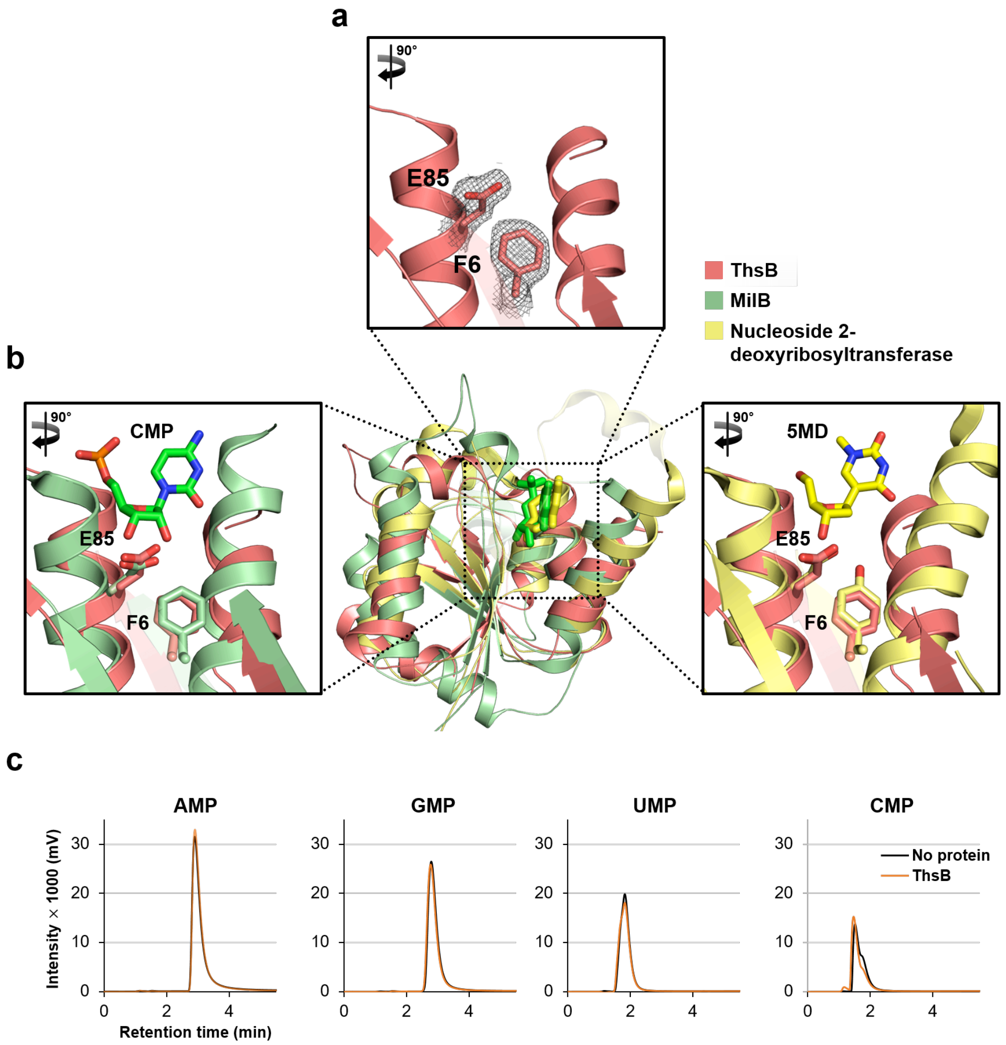

Supplementary Fig. 13. ThsB does not hydrolyze ribonucleoside monophosphates. a, Electron density map of key residues in the putative active site of ThsB. The $2mF_{obs} - DF_{calc}$ map is contoured at 1.0 σ. **b**, Structural comparison of ThsB and bacterial nucleotide hydrolases. ThsB (red) is structurally aligned with the CMP-bound MilB (PDB ID: 4JEM; green), a CMP hydrolase from *Streptomyces rimofaciens*, and 5-methyl-2′-deoxypseudouridine (5MD)-bound nucleoside 2-deoxyribosyltransferase (PDB ID: 1F8Y; yellow) from *Lactobacillus leichmannii*. The presence of Phe6 suggests that ThsB prefers ribosylated substrates if it is a nucleotide hydrolase. **c**, Activity assay of ThsB with ribonucleotides. Nucleotides such as AMP, GMP, UMP, and CMP were incubated with ThsB, and the reaction mixtures were analyzed using HPLC. The reactions in the presence of ThsB generated chromatograms essentially identical to those of the original nucleotides, indicating that ThsB did not hydrolyze them. Source data are provided as a Source Data file.

a

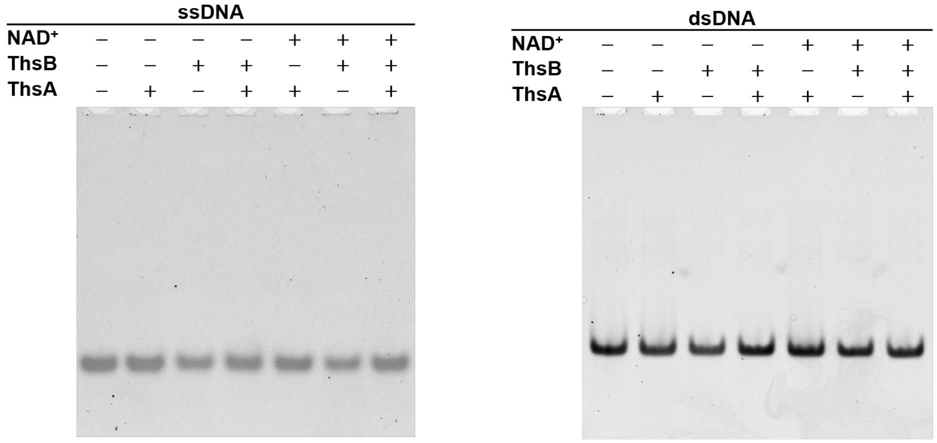

b

ssDNA	5′ –	CCTATTCACACATACTGGACAGCCAACTATGATAGATTAATAG	– 3′
dsDNA	5′ –	CCTATTCACACATACTGGACAGCCAACTATGATAGATTAATAG	– 3′
	3′ –	GGATAAGTGTGTATGACCTGTCGGTTGATACTATCTAATTATC	– 5′

Supplementary Fig. 14. EMSA for testing DNA binding of Thoeris proteins. a, EMSA with single-stranded and double-stranded DNAs. Thoeris proteins did not show DNA binding affinity with and without NAD^+. Source data are provided as a Source Data file. **b**, Sequences of DNAs used in the assay.

Supplementary References

1. Holm, L., Kaariainen, S., Rosenstrom, P. & Schenkel, A. Searching protein structure databases with dalilite v.3. *Bioinformatics* **24**, 2780-2781 (2008).
2. Krissinel, E. & Henrick, K. Inference of macromolecular assemblies from crystalline state. *J Mol Biol* **372**, 774-797 (2007).
